# Salinity-Induced Variation in Biochemical Markers Provides Insight into the Mechanisms of Salt Tolerance in Common (*Phaseolus vulgaris*) and Runner (*P. coccineus*) Beans

**DOI:** 10.3390/ijms17091582

**Published:** 2016-09-20

**Authors:** Mohamad Al Hassan, Mihaela Morosan, María del Pilar López-Gresa, Jaime Prohens, Oscar Vicente, Monica Boscaiu

**Affiliations:** 1 Instituto de Biología Molecular y Celular de Plantas, Universitat Politècnica de València-Consejo Superior de Investigaciones Científicas (UPV-CSIC), 46022 Valencia, Spain; moalhas@posgrado.upv.es (M.A.H.); mihaela.morosan@mail.com (M.M.); mplopez@ceqa.upv.es (M.d.P.L.-G.); ovicente@ibmcp.upv.es (O.V.); 2 Faculty of Horticulture, University of Agricultural Sciences and Veterinary Medicine (USAMV), 400372 Cluj-Napoca, Romania; 3 Instituto de Conservación y Mejora de la Agrodiversidad Valenciana, Universitat Politècnica de València (UPV), 46022 Valencia, Spain; 4 Instituto Agroforestal Mediterráneo, Universitat Politècnica de València (UPV), 46022 Valencia, Spain; mobosnea@eaf.upv.es

**Keywords:** ions concentration, *myo*-inositol, osmotic adjustment, *Phaseolus*, proline, salt tolerance

## Abstract

The evaluation of biochemical markers is important for the understanding of the mechanisms of tolerance to salinity of *Phaseolus* beans. We have evaluated several growth parameters in young plants of three *Phaseolus vulgaris* cultivars subjected to four salinity levels (0, 50, 100, and 150 mM NaCl); one cultivar of *P. coccineus*, a closely related species reported as more salt tolerant than common bean, was included as external reference. Biochemical parameters evaluated in leaves of young plants included the concentrations of ions (Na^+^, K^+^, and Cl^−^), osmolytes (proline, glycine betaine, and total soluble sugars), and individual soluble carbohydrates. Considerable differences were found among cultivars, salinity levels, and in their interaction for most traits. In general, the linear component of the salinity factor for the growth parameters and biochemical markers was the most important. Large differences in the salinity response were found, with *P. vulgaris* cultivars “The Prince” and “Maxidor” being, respectively, the most susceptible and tolerant ones. Our results support that salt stress tolerance in beans is mostly based on restriction of Na^+^ (and, to a lesser extent, also of Cl^−^) transport to shoots, and on the accumulation of *myo*-inositol for osmotic adjustment. These responses to stress during vegetative growth appear to be more efficient in the tolerant *P. vulgaris* cultivar “Maxidor”. Proline accumulation is a reliable marker of the level of salt stress affecting *Phaseolus* plants, but does not seem to be directly related to stress tolerance mechanisms. These results provide useful information on the responses to salinity of *Phaseolus*.

## 1. Introduction

Pulses (family Fabaceae) are important staple foods for a large part of the human population as they are a major source of protein, vitamins, minerals, and fiber [1]. In recognition of the relevance of pulses for mankind, 2016 has been declared as the International Year of Pulses. Among pulses, *Phaseolus vulgaris* L., the common bean, is cultivated all over the world, and is the most important legume for human nutrition [2]. *Phaseolus coccineus* L., the runner bean, is also cultivated worldwide at a much smaller scale than *P. vulgaris*, although locally it may be very important, like in Mexico where it is the second most important legume in the local diet after the common bean [3].

Adverse environmental conditions, mostly drought and soil salinity, are a major cause of crop losses in *Phaseolus* [4,5,6,7,8]. As all major crops, the common bean is a glycophyte, sensitive to salt, and even relatively low soil salinity levels (below 2 dS·m^−1^) significantly reduce crop productivity [4]. At a salinity equivalent to 100 mM NaCl, pod yield per plant in the common bean decreased by 85% [5], although some cultivars appear to be significantly more tolerant to salt stress than others [6,7,8]. Losses due to salinity are expected to increase in the near future—at least in arid and semiarid regions, where pulses are profusely grown—due to the forecasted effects of climate change [9], and subsistence farming in developing countries will be especially affected [10]. Therefore, an effective approach to increase beans’ crop yields over the next decades could be based on the selection of salt stress-tolerant cultivars [11].

One of the basic salt stress responses involves the control of ion homeostasis and the maintenance of cellular osmotic balance—water transport into the cell, compartmentalization of toxic ions in the vacuole, and synthesis and accumulation of compatible solutes or osmolytes in the cytoplasm—to counteract cellular dehydration caused by high soil salinity, but also by other stressful conditions such as drought, cold or high temperatures [12,13]. Osmolytes are involved in osmotic adjustment and also have osmoprotectant roles [14,15,16]. Comparative studies correlating stress responses with the relative tolerance of genetically related taxa—different species of the same genus, different varieties or cultivars of the same species—may be extremely useful to define those mechanisms that are most relevant for the tolerance to salinity in a given crop [17,18]. The present work focuses on the salt-induced accumulation of ions and osmolytes in the leaves as biochemical markers of the aforementioned basic responses to salinity, although many different physiological and biochemical responses of beans to salt stress have been previously investigated and reported, including a decrease in the uptake and total nitrogen levels [19], the reduction of stomatal conductance and photosynthetic capacity [20], the induction of oxidative stress and activation of some antioxidant enzymes [21], and the apparent decrease of catalase activity [22], to cite just a few of these studies.

We have analyzed the relative tolerance to salt stress during vegetative growth in plants of three *P. vulgaris* cultivars and in one cultivar of *P. coccineus*, a species that has been previously reported as being more stress-tolerant than the common bean [23]. Responses to stress are dependent on the plant developmental stage [24,25,26]; during the early stages of development, seedlings and young plants are generally more sensitive to stress than adult plants and it should be easier to detect differences in the relative tolerance of the investigated cultivars.

The major aim of our study was to obtain relevant information on the biochemical mechanisms underlying plant tolerance to salinity and, specifically, to establish which responses to salt stress are the most relevant for tolerance in *Phaseolus*. We assumed that differences in the stress tolerance of the selected cultivars could be explained by differences in the efficiency of the control of ion transport and maintenance of cellular osmotic balance.

## 2. Results

### 2.1. Electric Conductivity of Substrates

Electric conductivity (EC_1:5_) was recorded in samples of the pot substrates after three weeks of salt stress treatments (Table 1). The increase in EC_1:5_ was basically linear (Table 2), correlating with the concentration of the saline solutions used in the treatments. The variety x salinity interaction was nonsignificant, indicating no differences among varieties in the EC_1:5_ at the different concentrations studied, which shows that the substrate conditions for each salinity treatment were the same for all the varieties.

### 2.2. Effect of Salt Stress on Plant Growth

**Figure 1:**
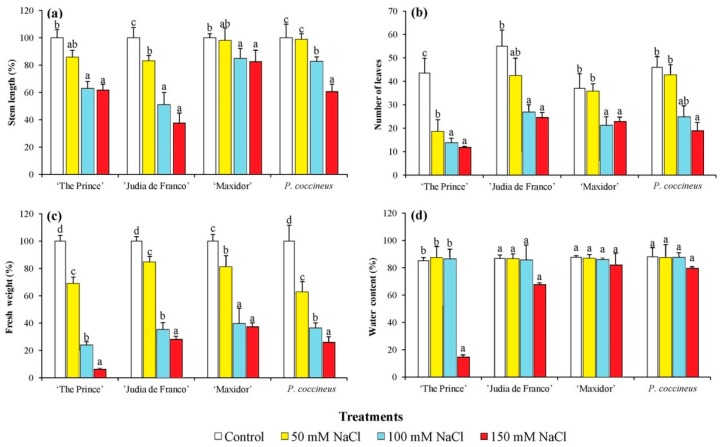
Salt stress-induced changes in growth parameters of 5-week-old *Phaseolus* plants. Salt stress-induced changes in: (**a**) stem length (%), with the mean stem lengths of control, non-treated plants (*Phaseolus vulgaris*, cv. “The Prince”: 60.00 cm; cv. “Judía de Franco”: 174.61 cm; cv. “Maxidor”: 44.16 cm; *Phaseolus coccineus*: 219.00 cm) considered as 100% for each cultivar; (**b**) number of leaves; (**c**) fresh weight (%), with the mean fresh weight of control plants (*Phaseolus vulgaris*, cv. “The Prince”: 31.54 g; cv. “Judía de Franco”: 34.87 g; cv. “Maxidor”: 17.17 g; *Phaseolus coccineus*: 30.26 g) considered as 100% for each cultivar; (**d**) water content (%). Measurements were performed after three weeks of treatment. The values shown are means with SD (*n* = 5). For each cultivar, different lowercase letters indicate significant differences between treatments according to the Tukey test (α = 0.05).

Compared to the controls, upon salt treatment the average stem length of the plants decreased in a mostly linear concentration-dependent manner, although the interaction variety × salinity, particularly the linear component, was significant indicating differences among varieties in the linear trend response to salinity (Table 2). At 150 mM NaCl concentration, the stem length was reduced by 40% in *P. coccineus* and *P. vulgaris* cultivar (cv.) “The Prince”, and by more than 60% in cv. “Judía de Franco”, but by only 20% in cv. “Maxidor” (Figure 1a).

The number of leaves also decreased with increasing salt concentrations, with some differences among cultivars (Figure 1b), but again the most important factor explaining the observed differences was the linear component of salinity (Table 2). “The Prince” appeared to be the cultivar most affected by salt, as the number of leaves had already dropped by more than 50% at the lowest concentration tested, 50 mM NaCl, and by approximately 75% in the presence of 150 mM NaCl, whereas the reduction of average leaf number in *P. coccineus* and *P. vulgaris* cv. “Maxidor” under the same conditions was much lower (Figure 1b).

For fresh weight (FW), the average differences among varieties and its interaction with salinity were very low (in both cases below 4% of the total sums of squares) compared to the differences among salinity treatments (above 92% of the total sums of squares), where the linear component was again the most important (Table 2). The greatest reduction of fresh mass of the aerial part of the plants was found in “The Prince”, with values of ca. 80% after treatment with 100 mM NaCl and 95% in the presence of 150 mM NaCl. The least affected by salt stress appeared to be “Maxidor”—about 63% decrease in FW in the 150 mM NaCl treatment—followed by “Judía de Franco”, and *P. coccineus* (Figure 1c).

In general, no significant reduction of water content was observed in the salt-treated plants. In this case, the greatest value for the contributions to the sums of squares was for the interaction between variety and salinity (Table 2), with high and significant values for the linear and quadratic components of the interaction. This was mostly caused by the difference in the performance of *P. vulgaris* “The Prince”, for which at the highest NaCl concentration tested (150 mM), a significant decrease in water content (nearly two-fold) was detected (Figure 1d).

### 2.3. Ion Contents

**Figure 2:**
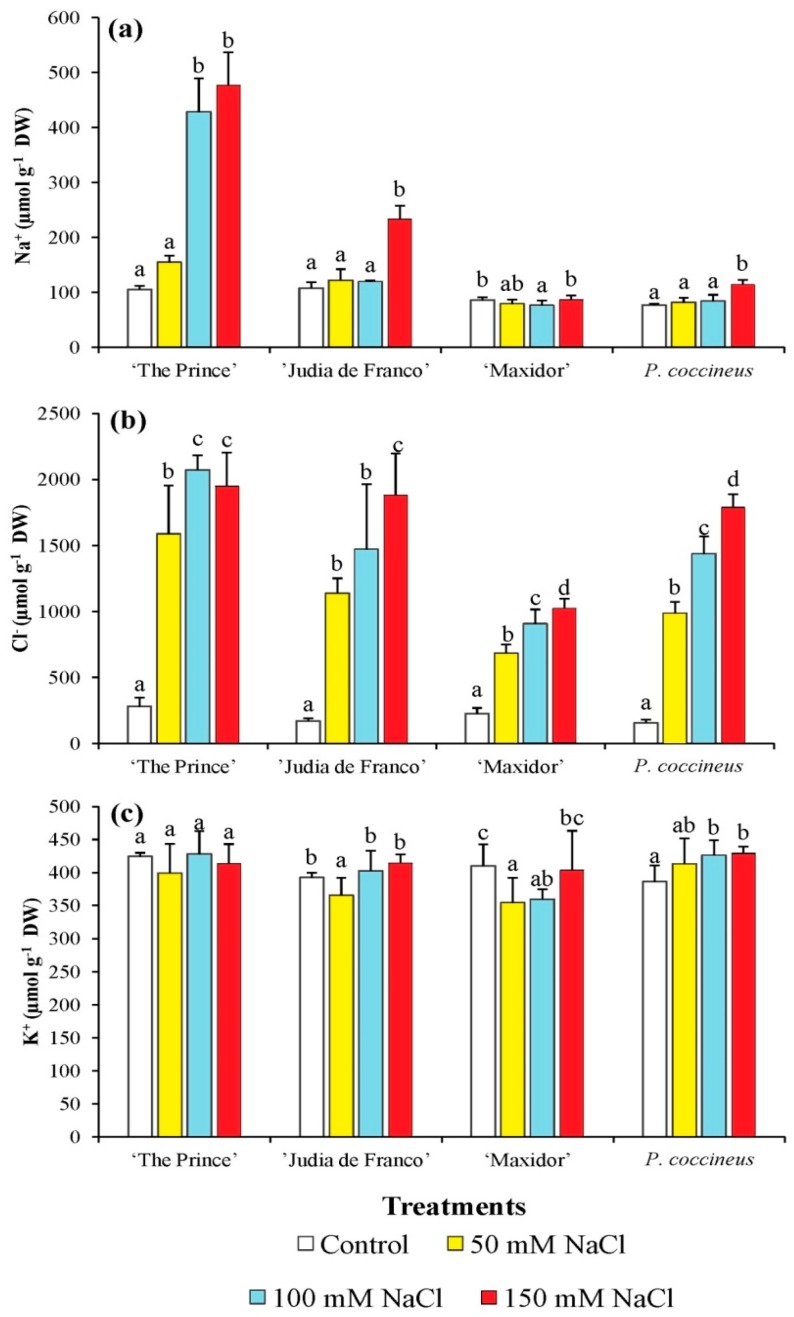
Salt stress-induced changes in ions levels (Na^+^, Cl^−^, and K^+^) of 5-week-old *Phaseolus* plants. Salt stress-induced changes in: (**a**) sodium, (**b**) chloride, and (**c**) potassium contents in leaves of *Phaseolus* plants of the studied cultivars. Measurements were performed after three weeks of treatment. The values shown are means with SD (*n* = 5). For each cultivar, different lowercase letters indicate significant differences between treatments according to the Tukey test (α = 0.05).

For Na^+^ concentration, very large differences among cultivars were found, with much more importance of varietal differences than salinity levels, which were mostly explained by a linear trend; however, very important effects were found for the interaction between variety and salinity (Table 3). The cultivars “The Prince” and “Judía de Franco”, and in particular the former, had much higher levels of Na^+^ than “Maxidor” and *P. coccineus* (Figure 2). Also, the patterns of sodium accumulation in the aerial part of the plants upon salt stress treatment were different for the *P. vulgaris* cultivars. Sodium levels rose four- to five-fold in the plants treated with 100 and 150 mM NaCl in “The Prince”—the most salt-sensitive cultivar, according to growth inhibition measurements—while a two-fold increase was measured in “Judía de Franco”, but only at the highest salt concentration. On the other hand, no statistically significant Na^+^ variation was detected in “Maxidor”, and in *P. coccineus* only a very small—but significant—increase in Na^+^ content was observed in the presence of 150 mM NaCl (Figure 2a). Contrarily to sodium, the salt concentration was much more important than the varietal factor in explaining the differences observed in Cl^−^ contents (Table 3). In this respect, although the interaction variety x salinity was significant, the four tested cultivars showed a similar qualitative pattern regarding chloride accumulation: in all of them Cl^−^ contents increased significantly, roughly in parallel with increasing external NaCl concentrations. In the presence of 150 mM NaCl, Cl^−^ levels rose up to 10- to 15-fold in *P. coccineus* and *P. vulgaris* “The Prince” and “Judía de Franco”, or about 5-fold in “Maxidor”, as compared to the control, non-treated plants (Figure 2b). Comparing the levels of both cations, it is clear that the salt-treated plants accumulated much more Cl^−^ than Na^+^ in their aerial parts, again with quantitative differences among cultivars: in the presence of high NaCl concentrations, Cl^−^/Na^+^ ratios of, approximately, 5, 10, 10, and more than 15 were calculated for “The Prince”, “Judía de Franco”, “Maxidor”, and *P. coccineus*, respectively (Figure 2a,b). Plant potassium contents showed little variation in response to increasing external salt concentrations in all cultivars (Figure 2c); as a consequence, most of the variation observed in the ANOVA was due to uncontrolled (residual) factors (Table 3). In this respect, although some significant differences were observed among cultivars, no differences were observed among salinity levels or for the interaction between variety and salinity (Table 3, Figure 2c).

### 2.4. Osmolyte Contents

**Figure 3:**
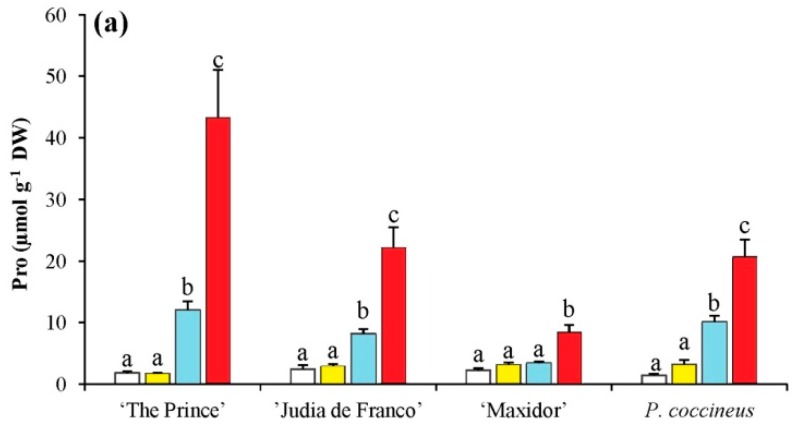

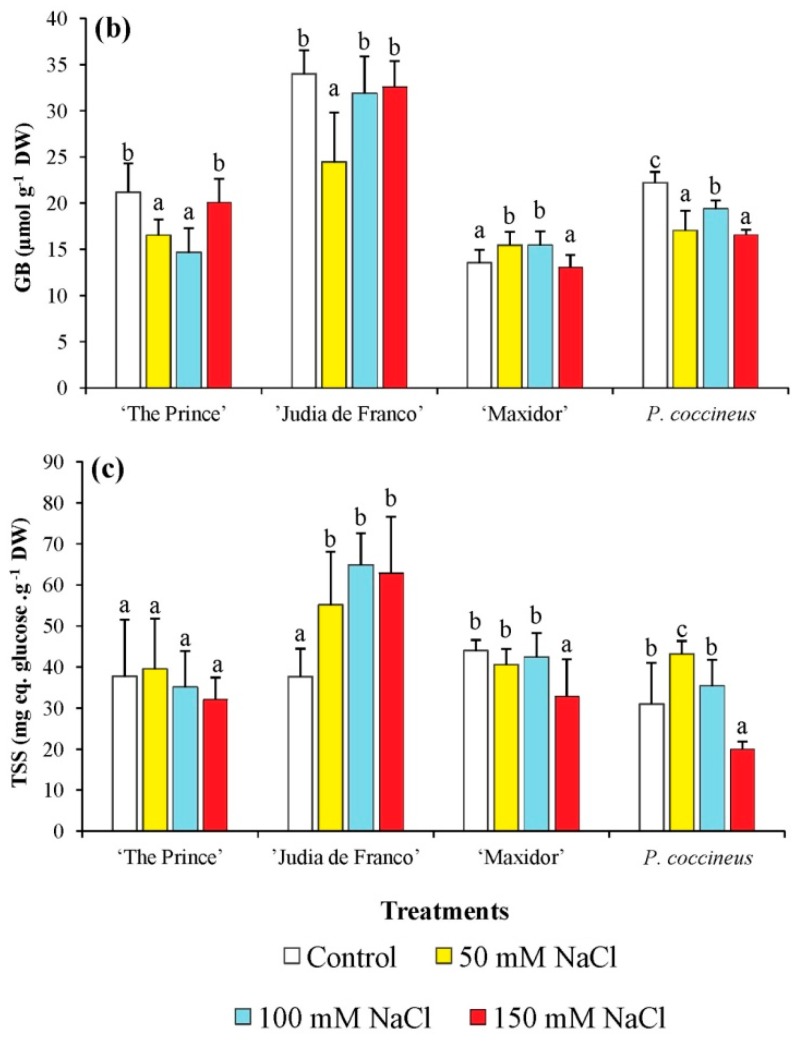
Salt stress-induced changes in the levels of osmolytes of 5-week-old *Phaseolus* plants. Salt stress-induced changes in the levels of: (**a**) proline (Pro); (**b**) glycine betaine (GB); and (**c**) total soluble sugars (TSS) in the same samples as in Figure 2. Measurements were performed after three weeks of treatment. The values shown are means with SD (*n* = 5). For each cultivar, different lowercase letters indicate significant differences between treatments according to the Tukey test (α = 0.05).

Proline (Pro) content was mostly affected by the salinity treatments, in particular by its linear component, and by the interaction between variety and the linear component of salinity (Table 3). The Pro levels in the control plants were relatively low and similar in all cultivars (2–4 µmol·g^−1^ dry weight (DW)), but increased significantly in the presence of 100 and 150 mM NaCl for all cultivars, although in “Maxidor” the increases were smaller. The highest Pro concentrations were reached in “The Prince”, with 12 and 42 µmol·g^−1^ DW in plants treated with 100 and 150 mM NaCl, respectively; the corresponding values for “Judía de Franco” and *P. coccineus* were 8 and 22 µmol·g^−1^ DW. However, in “Maxidor”, Pro levels at 150 mM NaCl reached a concentration of only 9 µmol·g^−1^ DW, much lower than in the other cultivars (Figure 3a).

For glycine betaine (GB) important differences were found among cultivars, which accounted for most of the variation observed (Table 3). Apparently, GB plays no role in responses to salt stress of the investigated *Phaseolus* cultivars (Figure 3b), since the salt-induced changes in the levels of this osmolyte were comparatively low (with just 5% of the sums of squares explained by salinity levels). The absolute GB concentrations were similar for *P. coccineus* and *P. vulgaris* “The Prince” and “Maxidor”, between 15 and 20 µmol·g^−1^ DW, and slightly higher in “Judía de Franco” (Figure 3b).

Mean values of total soluble sugars (TSS) were mostly affected by differences among varieties and for the interaction variety × salinity (Table 3). Here again, “Judía de Franco” presented slightly higher TSS levels under stress than those measured in the other studied cultivars (Figure 3c). TSS values did not show a clear pattern of variation in response to the salt treatments for any of the selected cultivars, and the observed differences, in general, were not statistically significant, possibly due in part to the variability in the TSS contents of individual plants, reflected in relatively large SD of the mean values (Figure 3c) and high contribution of the residual term to the sums of squares (Table 3).

**Figure 4:**
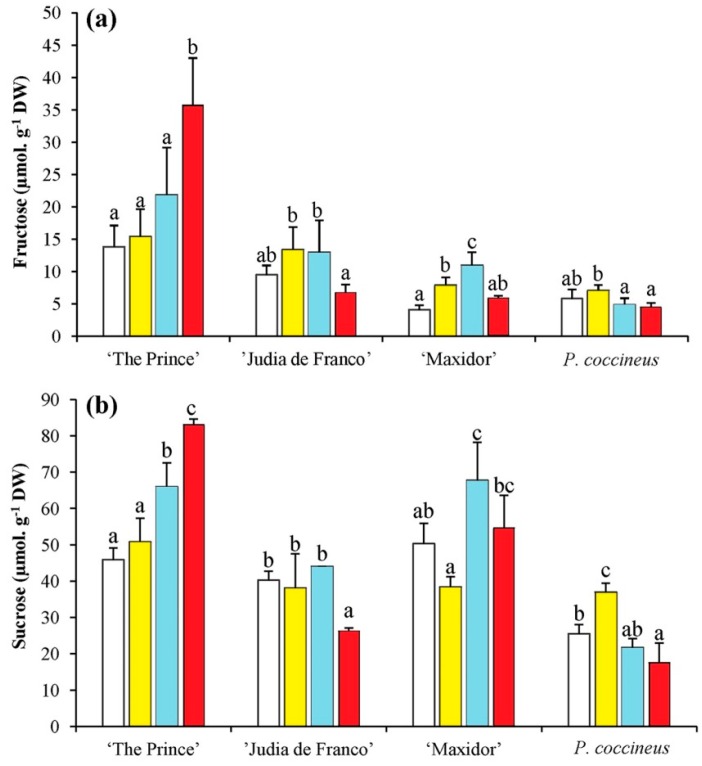

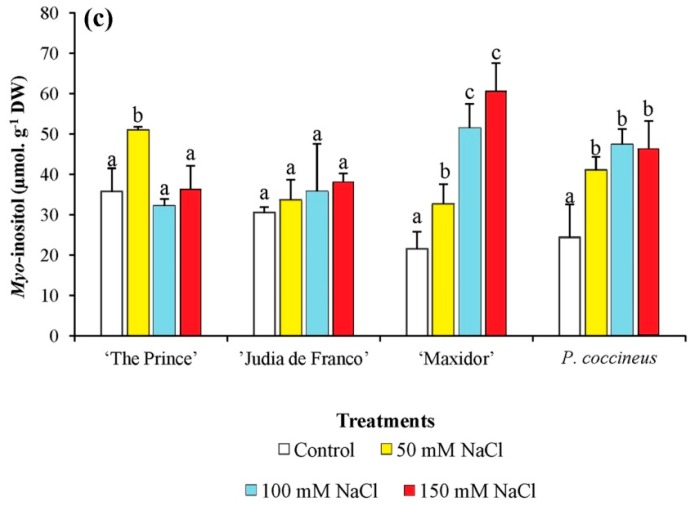
Salt stress-induced changes in the levels of major soluble carbohydrates of 5-week-old *Phaseolus* plants. Salt stress-induced changes in the levels of: (**a**) fructose; (**b**) sucrose; and (**c**) *myo*-inositol, separated by HPLC, in the same samples as in Figure 2. Measurements were performed after three weeks of treatment. The values shown are means with SD (*n* = 5). For each cultivar, different lowercase letters indicate significant differences between treatments according to the Tukey test (α = 0.05).

The major soluble carbohydrates in the extracts corresponded to fructose, sucrose, and *myo*-inositol. For fructose and sucrose, the differences among varieties were the greatest contributors to the sums of squares, followed by the interaction between variety and salinity—in particular with the linear component—and a low contribution for the factor salinity (Table 3). This indicates that the pattern of variation was different in the four analyzed cultivars. A clear salt-dependent increase in fructose and sucrose was only detected in “The Prince”, whereas in the other cultivars changes in the concentrations of both sugars were not statistically significant, or did not correlate with salinity levels (Figure 4a,b). Regarding *myo*-inositol, the differences caused by salinity levels (mostly the linear component) and, in particular, the variety × salinity interaction, presented the largest contribution to the observed differences (Table 3). A considerable increase of *myo*-inositol was detected in *P. vulgaris* “Maxidor” and, to a lesser extent, in *P. coccineus*, in parallel with increasing salt concentrations in the watering solution—about three-fold and two-fold, respectively, higher levels than in the non-stressed controls in the presence of 150 mM NaCl—but not in the other two studied varieties (Figure 4c).

## 3. Discussion

There are numerous papers reporting a wide range of salt tolerance in the genus *Phaseolus* [7,8], supporting the possibility of selecting the most tolerant genotypes to be used in breeding programs to improve this particular trait in beans. The decomposition of the salinity factor in its linear, quadratic, and cubic components and of the interaction of variety with the linear, quadratic, and cubic components of salinity revealed that, in general, the linear component was the most important, indicating that the range of salt concentrations used in the experiments (0–150 mM of NaCl in nutrient solution) was appropriate to study the response to salinity and perform early selection for tolerance to salinity in *Phaseolus* beans.

The most general effect of salt stress, and the easiest to quantify, is inhibition of growth, which allows plants to survive under adverse conditions by redirecting their resources (metabolic precursors and energy) from normal metabolism and growth to the activation of specific stress defense mechanisms [12,13]. Although salinity stress may affect the root-to-shoot growth ratio [27]—due to enhanced root growth in order to find soil areas with lesser salt concentration—in our case, the growth in pots with substrate and watering with a uniform saline solution would have minimized this potential effect. Determination of several aerial part growth parameters allowed us to establish the relative degree of tolerance to salt stress of the investigated bean varieties. *Phaseolus* *vulgaris* “The Prince” was the most sensitive, followed by “Judía de Franco”, while “Maxidor” was the most stress-tolerant of the selected common bean cultivars—even more than the *P. coccineus* cultivar tested, although this species has been previously reported as being more tolerant than *P. vulgaris* [23,28]. Although different plant sizes could theoretically contribute to differences in the tolerance to salinity, this does not seem to be the case for the selected cultivars, since the most salt-sensitive (“The Prince”) and the most tolerant (“Maxidor”) had similar stem length in the control plants and very different patterns of ion accumulation.

The comparative analysis of different genotypes, such as that reported here for several *Phaseolus* cultivars, may help to identify relevant mechanisms of tolerance, correlating their relative degree of tolerance to salt stress with changes in the levels of stress markers associated to specific responses. Our results indicate that one of the main reasons behind salt tolerance in *Phaseolus* is the presence of mechanisms that restrict the transport of Na^+^ to the aerial part of the plants, mechanisms that are more efficient in the relatively more tolerant cultivars. It is known that plants of the genus *Phaseolus* are able to exclude sodium from the shoots, even in the presence of relatively high NaCl concentrations in the soil [29]. Earlier autoradiography studies on *P. vulgaris* indicated that Na^+^ is retained in roots by binding at sites in the stele or at those bordering it [30]. This was confirmed later using X-ray microanalyses in *P. coccineus*, which also excluded Na^+^, but not Cl^−^ [31]. In more recent studies, a higher Na^+^ concentration in roots than in leaves was found in *P. vulgaris* and *P. latifolius*, showing that, within this genus, the basic mechanism to minimize the deleterious effects of sodium accumulation in the leaves is reducing transport of the ion from the roots [32]. The anion Cl^−^, on the other hand, displays high mobility within the plant and is not effectively compartmentalized in cells: chloride concentration was high in both the vacuole and the chloroplast-cytoplasm in salt-stressed plants of *P. vulgaris* [29]. Salt-sensitive *Phaseolus* genotypes gave a higher Cl^−^ concentration in leaves than more tolerant ones [32]. In all cultivars selected for the present study, Na^+^ concentration in leaves was clearly lower than that of Cl^−^, under the same external conditions, thus confirming this mechanism. Yet, the pattern of ion accumulation in response to increasing NaCl concentration in the nutrient solution varied, according to the relative salt tolerance of the different cultivars, with the lowest ion contents measured in the most tolerant cultivar (“Maxidor”), and the highest in the most sensitive (“The Prince”). Similar results, regarding accumulation of toxic ions in the aerial part of the plants, have been reported when comparing different cultivars of species from other Fabaceae genera. Relatively lower Na^+^ and Cl^−^ contents have been measured in the most tolerant cultivars, for example in chickpea [33], soybean [34], or pea [35].

Since Na^+^ can compete with K^+^ for the same transporters [12], mechanisms able to maintain relatively low Na^+^/K^+^ ratios would therefore contribute to salt tolerance. Increased K^+^ in foliar tissue upon salt treatments has been reported in beans by Seeman and Critchley [29], while other authors observed similar values in plants at moderate salinity levels and in the controls [32]. These latter authors also reported reduced K^+^ contents of salt-treated plants, and explained the differences in the distribution of this cation in the plants by the translocation of K^+^ from roots and stems to leaves due to activation of highly selective K^+^ transporters. The levels of K^+^ in leaves were similar for all four *Phaseolus* cultivars tested here, and did not vary significantly in the presence of salt; therefore, Na^+^/K^+^ ratios in leaves were dependent on Na^+^ contents, and were lower in the more tolerant than in the more sensitive cultivars.

Proline (Pro) seems to be a reliable marker of stress in *Phaseolus*, but the correlation between Pro accumulation and stress tolerance remained unclear. Higher free Pro levels have sometimes been reported in more salt-tolerant bean cultivars than in less tolerant ones [36]. However, there are also opposite reports of relatively higher Pro levels in more sensitive cultivars: in *P. vulgaris* [37], as well as, for example, in *Glycine max* [38]. In other plant species of the Fabaceae family, such as *Cicer arietinum* [39] or *Pisum sativum* [35], although Pro contents increased in response to increasing salinity, no correlation between Pro accumulation and the relative tolerance of different cultivars has been found, and the same has been reported for plants of other families [40,41,42]. Our experimental approach should help to clarify the confusion often found in the literature between the concepts of “stress responses” and “stress tolerance”. The present study revealed that Pro accumulation is a common response to salt stress in the four cultivars analyzed here, in agreement with the aforementioned published results. Yet, Pro cannot contribute significantly to their stress tolerance, since the levels reached in “Maxidor”, the most tolerant variety, were by far lower than in the other cultivars. In this case, Pro should be considered as a marker of the level of stress affecting the plants, and these results simply reflect the fact that “Maxidor” plants were less stressed than the others.

There are only a handful of references describing the presence of glycine betaine (GB) in *Phaseolus* [43,44], although at concentrations lower than those reported here, which are in turn much lower than the GB levels recorded in real GB accumulator species [45,46]. However, the applied salt stress treatments did not lead to significant increases of GB contents in the analyzed *Phaseolus* varieties.

Regarding soluble sugars, there are several publications dealing with the variation of soluble carbohydrate contents in beans under stress conditions [47,48,49], but it has been recently reported that salt stress-induced sugar accumulation in the genus *Phaseolus* barely contributes to the leaf osmotic potential [32]. We did not detect significant changes, correlated with the stress treatments, in the levels of total soluble sugars in the analyzed *Phaseolus* cultivars. However, after separation of the carbohydrate fraction by HPLC, strong increases in *myo*-inositol contents in the presence of NaCl were observed in cv. “Maxidor”, the most tolerant cultivar of *P. vulgaris*, and to a lesser extent also in *P. coccineus*. Therefore, this polyalcohol appears to play a significant role in the salt tolerance mechanisms in *Phaseolus* taxa, as it has been reported in other genera. Increases of *myo*-inositol levels in response to salt stress treatments have been detected in several species, such as kiwi [50], ice plant [51] or, among legumes, in chickpea [52]. It has also been shown that increasing the endogenous levels of *myo*-inositol, by transformation with appropriate biosynthetic genes, improved salt tolerance in transgenic plants of different species ([18] and references therein), thus supporting its functional role in the mechanisms of salt tolerance in plants. Publications on the presence of *myo*-inositol in *Phaseolus* species are, however, scarce; for example, glucose and inositol have been reported as the major protectant sugars in salt stressed beans [53], although we did not detect a significant variation of glucose in the cultivars analyzed here.

Apart from helping to identify the most relevant mechanisms of salt tolerance in beans, our experimental approach could be applied in breeding programs to improve salt tolerance of beans. It provides a rapid and simple method to screen a large number of varieties for an initial selection of the most resistant ones, eliminating the most salt-sensitive. Promising cultivars selected at the vegetative growth phase will have to be further analyzed at later developmental stages, since it is not possible to predict that they will also give higher yields under salt stress conditions.

## 4. Materials and Methods

### 4.1. Plant Material

Three varieties of *P. vulgaris*, (“The Prince”, “Judía de Franco”, and “Maxidor”), and one variety of *P. coccineus* (commercial cultivar “Moonlight”) were used in the present study. “The Prince” is a dwarf French bean cultivar, with a bushy growth, one of the most commonly used in Europe. “Judía de Franco” is a local landrace of *P. vulgaris* from the province of Teruel (Spain) with indefinite growth. “Maxidor” is a dwarf French bean cultivar with a bushy growth and precocious flowering. “Moonlight” is a *P. coccineus* cultivar originating from Mexico. Seeds of “The Prince” and “Maxidor” were purchased from AGROSEM IMPEX (Targu Mures, Romania), those of “Judía de Franco” were obtained from the Germplasm Bank of COMAV (Institute for Conservation and Improvement of Valencian Agrodiversity, Universitat Politècnica de València), and “Moonlight” *P. coccineus* seeds were obtained from Thompson and Morgan, AJP Garden and Crafts (Bristol, UK).

### 4.2. Growing Conditions and Stress Treatments

Seeds were sterilized with a 0.3% (*v*/*v*) solution of sodium hypochlorite for 5 min, rinsed in distilled water and then sown on a moistened mixture of peat, perlite, and vermiculite (2:1:1) in 0.5 L pots (diameter = 11 cm) placed in plastic trays (12 pots per tray). One seed was placed per pot on the substrate surface. Throughout the germination process, the substrate was kept moderately moistened using half-strength Hoagland nutrient solution [54]. Salt stress treatments (50, 100, and 150 mM NaCl) were started 16 days after germination, when the first trifoliate leaves had already appeared. The control plants (0 mM NaCl) were watered twice a week with half-strength Hoagland nutrient solution added to the trays (1 L per every three plants; that is, 4 L per tray). For the salt stress treatments, the plants were watered with the same volume of nutrient solution but containing NaCl at the final concentrations indicated above. All the experiments were conducted for three weeks, selecting five plants per treatment, in an environment chamber under the following controlled conditions: long day photoperiod (16 h of light and 8 h of darkness), temperature of 23 °C during the day and 17 °C at night. Relative humidity ranged between 50%–80% while all the treatments were underway.

### 4.3. Soil Analysis

The electrical conductivity (EC_1:5_) of the substrate was checked at the end of the treatments. Soil samples were taken from the five pots selected per treatment, air-dried and then passed through a 2 mm sieve. A soil:water (1:5) suspension was prepared in distilled water and stirred for 1 h at 600 rpm and 21 °C. EC was measured with a Crison Conductivity meter 522 (Crison Instruments SA, Barcelona, Spain) and expressed in dS·m^−1^.

### 4.4. Plant Growth Parameters

After three weeks of salt treatment, the aerial part of each plant was collected and the following growth parameters were determined: stem length, leaf number, fresh and dry weight, and percentage of water content. Fresh material was stored at −20 °C for further studies. Stem length and fresh weight were expressed as percentage of the values corresponding to the non-stressed controls. To determine water content, part of each sample was weighed (FW), dried at 65 °C until constant weight (48–72 h), and then reweighed (DW); water content of each sample (%) was calculated as described in [55].

### 4.5. Ion Content Measurements

Monovalent ion contents after the stress treatments were determined according to a previously published procedure [56] in aqueous extracts obtained by heating the samples (0.15 g of dried, ground plant material in 25 mL of water) in a water bath, for 1 h at 95 °C, followed by filtration through filter paper (particle retention 8–12 µm). Sodium and potassium were quantified with a PFP7 flame photometer (Jenway Inc., Burlington, VT, USA) and chlorides were measured using a Merck Spectroquant Nova 60^®^ spectrophotometer and its associated test kit (Merck, Darmstadt, Germany).

### 4.6. Osmolyte Quantification

Proline (Pro) was extracted with 3% (*w*/*v*) sulfosalicylic acid, from 0.2 g of frozen plant material in liquid nitrogen, and was quantified according to the acid–ninhydrin method [57]. Pro concentration was expressed as µmol·g^−1^ DW. Glycine betaine (GB) was determined from 0.1 g dry material as previously described [58]. GB concentration was expressed as µmol·g^−1^ DW. Total soluble sugars (TSS) were measured in 0.1 g dry plant material suspended in 3 mL 80% (*v*/*v*) methanol, following the phenol/sulphuric acid method of Dubois et al. [59]. TSS contents were expressed as “mg equivalent of glucose” per g DW.

### 4.7. HPLC Analysis of Carbohydrates

The soluble sugar fraction (mono- and oligosaccharides) was analysed using a Waters 1525 high performance liquid chromatography coupled to a 2424 evaporative light scattering detector (ELSD). The source parameters of ELSD were the following: gain 75, data rate 1 point per second, nebulizer heating 60%, drift tube 50 °C, and gas pressure 2.8 kg·cm^−2^. The analysis was carried out injecting 20 µL aliquots of the samples with a Waters 717 autosampler into a Prontosil 120-3-amino column (4.6 mm × 125 mm; 3 µm particle size). An isocratic flux (1 mL/min) of 85% “acetonitrile” (J.T. Baker-Avantor Performance Materials, Deventer, The Netherlands) during 25 minutes was applied in each run. Standards of glucose, fructose, sucrose, and *myo*-inositol were used to identify peaks by coinjection. Sugars were quantified with peak integration using the Waters Empower software and comparison with glucose, fructose, sucrose, and *myo*-inositol standard calibration curves.

### 4.8. Statistical Analysis

Data were analyzed using Statgraphics Centurion XVI (Statgraphics-Statpoint Technologies, Inc., Warrenton, VA, USA) software package. Before the analysis of variance, the Shapiro–Wilk test was used to check for validity of normality assumption and Levene’s test for the homogeneity of variance. Two-way ANOVA tests with variety (V), salinity (S), and V × S interactions were performed. Whenever the salinity factor and the V × S interaction were significant, the orthogonal decomposition of the sums of squares in the linear, quadratic, and cubic components of the factor salinity and of the interaction of the factor variety with these three components of the salinity was performed using a trend analysis [60]. The significance of the differences among salinity levels for each variety was tested by one-way ANOVA at a 95% confidence level and post hoc comparisons were made using the Tukey HSD test. All means throughout the text are followed by the SD value.

## 5. Conclusions

We have determined the relative tolerance to salt stress of four *Phaseolus* cultivars during the early stage of vegetative growth. Tolerance was highest in *P. vulgaris* “Maxidor”, followed by the *P. coccineus* cultivar tested (“Moonlight”), while the cultivar most sensitive to stress appears to be *P. vulgaris* “The Prince”. Changes in the levels of ions and several osmolytes revealed that in conditions of high soil salinity, tolerance to stress in *Phaseolus* is mostly based on restriction of Na^+^ (and, to a much lesser extent, also of Cl^−^) transport to shoots, and on the accumulation of *myo*-inositol as the major functional osmolyte. This new information on biochemical traits in young plants of *Phaseolus* beans subjected to salinity stress contributes to understanding the mechanisms of salt tolerance in beans and to improving the efficiency in early selection of salinity tolerant varieties in this important pulse crop.

## Figures and Tables

**Table 1:** Electric conductivity (EC_1:5_, dS·m^−1^) of the substrates after 3 week treatments of the *Phaseolus* plants with the indicated NaCl concentrations. The values shown are means with SD (*n* = 20). Different superscript letters indicate significant differences between substrates experiencing different treatments according to the Tukey test (α = 0.05).

Treatment	Substrate EC_1:5_ (dS·m^−1^)
Control	0.60 ± 0.18 ^a^
50 mM NaCl	1.89 ± 0.33 ^b^
100 mM NaCl	2.61 ± 0.28 ^c^
150 mM NaCl	3.55 ± 0.63 ^d^

**Table 2:** Percentage of the total sum of squares for the effects of variety (V), salinity (S), and their interaction (V × S) for the substrate electrical conductivity and plant growth parameters of four *Phaseolus* varieties grown at four salinity levels (0, 50, 100, and 150 mM of added NaCl to the medium solution). For the S and V × S sources of variation, the sum of squares has been decomposed in the linear, quadratic, and cubic components.

Source of Variation	Degrees of Freedom	Electrical Conductivity (dS·m^−1^)	Stem Length (%)	Number of Leaves	Fresh Weight (%)	Water Content (%)
Variety (V)	3	0.84 ^ns^	19.48 ****	17.73 ***	3.22 ****	16.24 ****
Salinity (S)	3	88.78 ****	59.21 ****	64.77 ***	92.55 ****	17.33 ****
Linear	1	87.64 ****	57.52 ****	61.33 ***	88.32 ****	10.85 ****
Quadratic	1	0.56 *	0.01 ^ns^	1.95 ***	1.55 ****	5.80 ***
Cubic	1	0.59 *	1.67 **	1.49 **	2.68 ****	0.68 ^ns^
V × S	9	1.77 ^ns^	11.76 ****	8.42 ***	2.80 ****	42.15 ****
Linear	3	–	8.80 ****	2.96 ***	1.62 ****	23.15 ****
Quadratic	3	–	2.60 **	2.86 ***	0.32 **	16.78 ****
Cubic	3	–	0.36 ^ns^	2.60 **	0.86 ****	2.22 ^ns^
Residual	64	8.61	9.55	9.08	1.42	24.28

^ns^, *, **, ***, **** indicate non-significant or significant at *p* < 0.05, *p* < 0.01, *p* < 0.001, or *p* < 0.0001, respectively.

**Table 3:** Percentage of the total sum of squares for the effects of variety (V), salinity (S), and their interaction (V × S) for the ions, osmolytes, and carbohydrates contents in the leaves of plants of four *Phaseolus* varieties grown at four salinity levels (0, 50, 100, and 150 mM NaCl added to the medium solution). For the S and V × S sources of variation, the sum of squares has been decomposed in the linear, quadratic and cubic components.

Source of Variation	Degrees of Freedom	Na	Cl	K	Proline	Glycine Betaine	Total Soluble Sugars	Fructose	Sucrose	*Myo*-Inositol
Variety (V)	3	46.51 ****	17.03 ****	14.12 **	9.70 ****	75.18 ****	30.49 ****	55.59 ****	60.75 ****	5.11 **
Salinity (S)	3	18.89 ****	69.32 ****	6.75 ^ns^	66.45 ****	5.00 ****	6.57 *	5.71 ****	5.01 ****	32.47 ****
Linear	1	17.99 ****	62.19 ****	–	53.09 ****	0.89 *	0.02 ^ns^	4.49 ****	3.10 ****	28.74 ****
Quadratic	1	0.58 *	6.83 ****	–	12.95 ****	2.52 ****	6.54 ***	0.75 ^ns^	0.70 *	2.41 **
Cubic	1	0.32 ^ns^	0.30 ^ns^	–	0.41 *	1.59 ***	0.00 ^ns^	0.47 ^ns^	1.21 **	1.32 *
V × S	9	28.88 ****	7.48 ****	12.53 ^ns^	20.03 ****	11.50 ****	27.98 ****	24.88 ****	27.54 ****	41.24 ****
Linear	3	24.17 ****	4.51 ****	–	15.88 ****	0.77 ^ns^	23.16 ****	16.93 ****	18.60 ****	29.53 ****
Quadratic	3	0.64 ^ns^	2.92 ****	–	4.06 ****	6.47 ****	3.56 ^ns^	7.44 ****	3.02 ****	1.29 ^ns^
Cubic	3	4.07 ****	0.05 ^ns^	–	0.09 ^ns^	4.26 ****	1.26 ^ns^	0.51 ^ns^	5.91 ****	10.42 ****
Residual	64	5.72	6.16	66.60	3.82	8.32	34.96	13.81	6.70	21.18

^ns^, *, **, ***, **** indicate nonsignificant or significant at *p* < 0.05, *p* < 0.01, *p* < 0.001, or *p* < 0.0001, respectively.
